# Left Inferior Vena Cava With Infrahepatic Disruption, Azygos Continuation, and Retroaortic Left Renal Vein – A Complex, Symptomatic Caval Anomaly Managed With Endovascular Intervention

**DOI:** 10.1177/15385744231171199

**Published:** 2023-04-21

**Authors:** Andrew Samoyedny, Ryan Cobb

**Affiliations:** 21798 Hospital of the University of Pennsylvania, Philadelphia, PA, USA

**Keywords:** Left inferior vena cava, azygos continuation, infrahepatic disruption, retroaortic, congenital anomaly, pelvic venous disease, endovascular, treatment, miscarriage

## Abstract

**Background:** Complex congenital anomalies of the inferior vena cava (IVC) are rare sequelae of inappropriate persistence or regression of embryological precursor veins. These anomalies are typically asymptomatic and generally do not warrant intervention. **Case Presentation:** Here we present a case of severely symptomatic left IVC with infrahepatic disruption, azygos continuation, and retroaortic left renal vein causing symptoms of severe pelvic congestion and recurrent miscarriages (8 total) in a 41 year old female. The patient was treated with stenting of the compressed retroaortic portion of the IVC/left renal vein. Four months post-procedure, the stent remained patent and the patient reported considerable improvement in their venous congestion symptoms. Most notably, as of the writing of this report, the patient is 38 weeks pregnant. **Conclusions:** The case is notable for its severe symptomatology of pelvic venous disease including recurrent miscarriage. More importantly, it represents the first documented case of successful retroaortic endovascular management of such a venous anomaly, in which the entirety of the typical IVC drainage occurred via a compressed left-to-right retroaortic crossover.

## Introduction

The inferior vena cava (IVC) is composed of 4 segments with distinct paired embryological precursor veins: an infrarenal segment (right supracardinal vein), renal segment (right sub-supracardinal anastomoses), suprarenal segment (right subcardinal vein), and hepatic segment (right vitelline vein). Inappropriate regression or persistence of these paired venous precursors in the sixth-eighth weeks of life produces congenital anomalies of the IVC, which include but are not limited to: left IVC, duplicated IVC, azygos/hemiazygos continuation of the IVC (with interruption of the hepatic segment), and circumaortic or retroaortic renal veins. When isolated, these anomalies are rare, typically asymptomatic, and thought to range in prevalence from .2% to .6%.^1,2^ When combined to form complex IVC anomalies, they are rarer still and may predispose patients to manifestations of chronic venous disease. Here, we present a rare symptomatic complex IVC anomaly consisting of a left IVC with midline crossover via a retroaortic left renal vein (LRV), azygos continuation, and complete disruption of the infrahepatic IVC.

## Case Report

**Figure 1.**
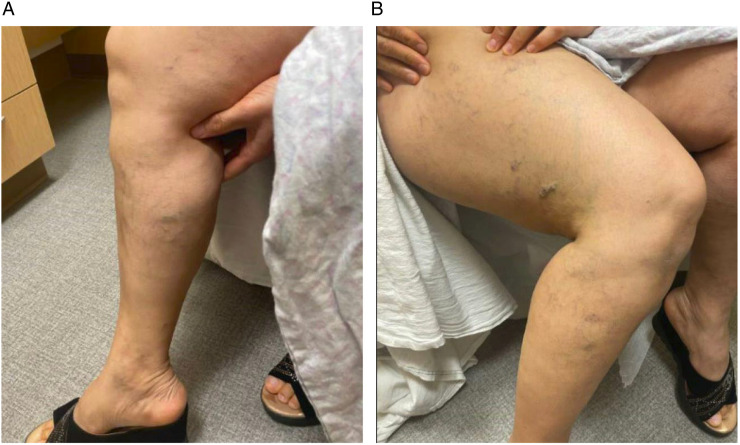
(A, B) presenting symptoms included lower extremity swelling, pain, varices of the calf and thigh. Additional varices of the vulva, vagina and abdominal wall (not pictured) were observed.

**Figure 2.**
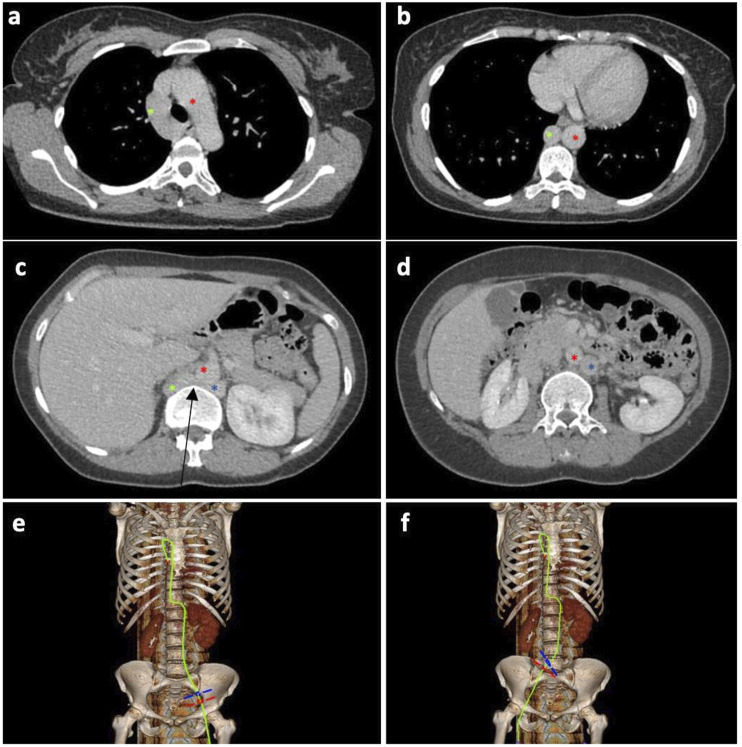
(A-D) Axial contrast enhanced CT displaying azygos continuation (green asterisk) of the left IVC (blue asterisk) with compression of the left renal vein (black arrow) between the spine and aorta (red asterisk). 2(E-F) 3-D reconstruction of the course of venous drainage from the bilateral lower extremities.

**Figure 3.**
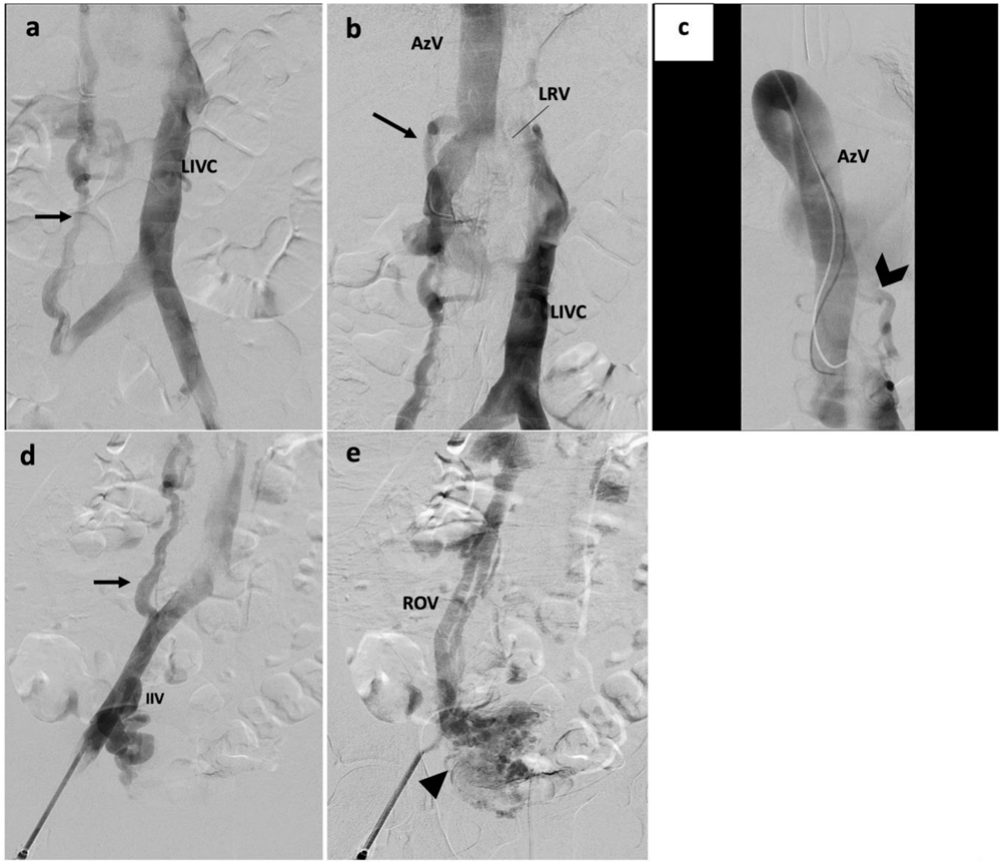
(A) Pre-intervention venography demonstrating a left IVC with dominant right ascending lumbar vein (black arrow) draining into the azygos vein. 3(B) Left IVC crosses midline via a compressed retroaortic left renal vein (black arrow) drains into the azygos vein. 3(C) Enlarged azygos vein drains into the right brachiocephalic vein. Collateral flow from the left common iliac and renal veins occurs via left paraspinal veins (black chevron). 3(D) A dominant ascending lumbar vein (black arrow) arises from the right common iliac vein. There is reflux into the right internal iliac vein (IIV). 3(E). Right parauterine varicosities are seen (arrowhead), as well as an antegrade draining right ovarian vein (ROV).

Fourty one-year-old female with history of bilateral lower extremity swelling, pain, parasthesias, varices of the legs, thighs (Figure 1), vulva, vagina, and abdominal wall, severe pelvic pain, dyspareunia, dysmenorrhea, and 8 miscarriages (CEAP classification: C_3_E_c_A_d_P_o_; Pelvic Venous Disease SVP classification: S_3b_V_3b_P_LRVOC_). Venography, intravascular ultrasound (IVUS), and cross-sectional imaging revealed left IVC with retroaortic LRV continuing as a dilated azygos vein terminating in the right brachiocephalic vein and complete disruption of the infrahepatic IVC. Compression of the retroaortic portion of the LRV resulted in severe/near occlusive stenosis. Collateral flow from the left common iliac and renal veins was via paraspinal veins arising from the LRV and a dominant right ascending lumbar vein draining into the azygos vein. Drainage of the right lower extremity was via the right ascending lumbar vein and parauterine varicosities draining into a hypertrophied right ovarian vein terminating in the right renal vein (Figures 2, 3). No microscopic hematuria was present to suggest a posterior nutcracker syndrome.

Open surgery was considered and transplant surgery consulted, given the possibility of endovascular hardware failure and possible diaphragmatic irritation by the stent. Renal vein transposition was determined to be too high risk given the presence of variant anatomy and the possibility of expected abdominal surgical complications including bowel obstruction due to adhesions, infection, vascular or solid organ injury, hemorrhage, and death.

**Figure 4.**
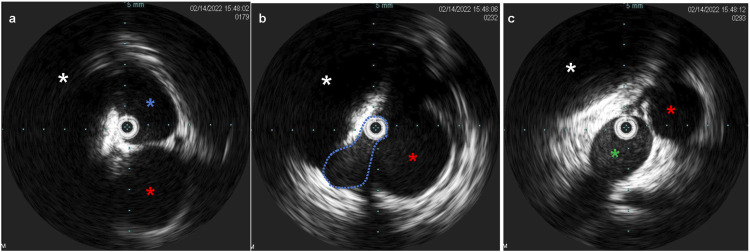
(A) Intravascular ultrasound (IVUS) demonstrates a patent infrarenal left IVC (blue asterisk) adjacent to the aorta (red asterisk). (B) IVUS at the retroaortic IVC/renal vein segment demonstrates compression of the venous lumen (outline in blue) behind the aorta (red asterisk) against the spine (white asterisk). (C) Above the retroaortic segment, the IVC continues as the patent enlarged azygos vein (green asterisk), adjacent to aorta (red asterisk) within the thorax.

**Figure 5.**
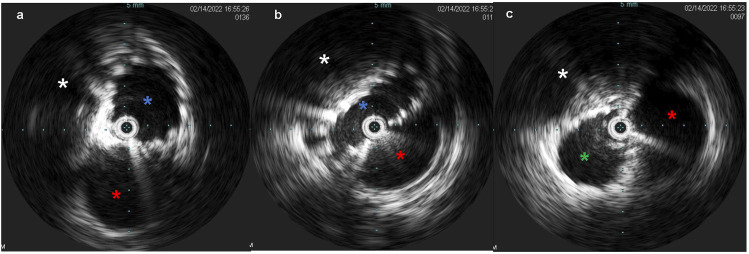
(A) Post-intervention IVUS demonstrates good apposition of the stent to the LIVC wall (blue asterisk) proximal to the retroaortic segment, with adjacent aorta (red asterisk). (B) Post-intervention IVUS demonstration the stented retroaortic venous segment (blue asterisk) posterior to aorta (red asterisk), with compression against the spine (white asterisk). (C) Post-intervention IVUS demonstrates good apposition of the stent to the wall of the azygos vein (green asterisk), with adjacent aorta (red asterisk).

**Figure 6.**
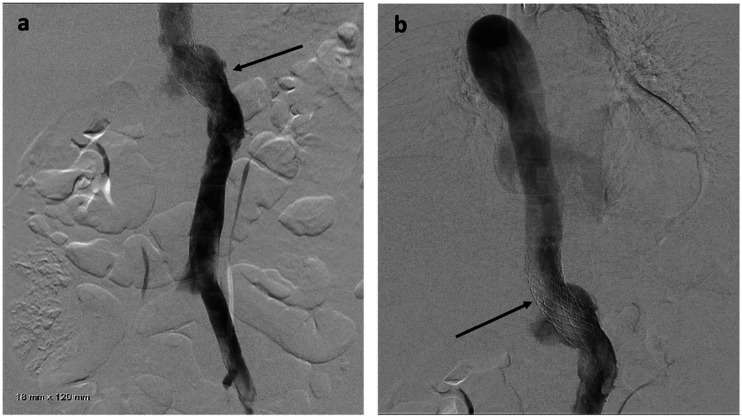
(A-B) Post-intervention venography demonstrates a patent and stented (black arrow) left IVC, retroaortic left renal vein, and proximal azygos vein with interval resolution of the prominent right lumbar and left paraspinal collateral veins.

Venous access was obtained with a 5F 10 cm right internal jugular venous sheath and a 10F 10 cm left common femoral venous sheath. Via the left common femoral access, pre-intervention venography was performed. IVUS (Figure 4) demonstrated an average luminal diameter of 13.7 mm 1 cm inferior to the stenosis and 14.7 mm 1 cm above the stenosis, thus guiding the selection of an 18 mm diameter stent – slightly oversized given the presence of compression. An 18 mm × 120 mm Abre™ self-expanding venous stent (Medtronic, Dublin, Ireland) was placed, traversing the left IVC, the retroaortic LRV, and the inferior portion of the dilated azygos vein with post-deployment dilation utilizing an 18 mm × 40 mm angioplasty balloon. Post-stenting IVUS was performed (Figure 5), confirming good apposition of the stent to the venous wall**.** Post intervention venogram demonstrated venous drainage of the bilateral lower extremities via the left IVC into the left renal and azygos veins with absence of previously noted collaterals (Figure 6). Coiling of the refluxing ovarian vein was deferred pending post-procedural course and imaging. She was discharged with prescriptions for apixaban 5 mg by mouth twice daily and clopidogrel 75 mg by mouth daily for 6 weeks (until follow-up), at which point she was transitioned to daily baby aspirin (81 mg).

**Figure 7.**
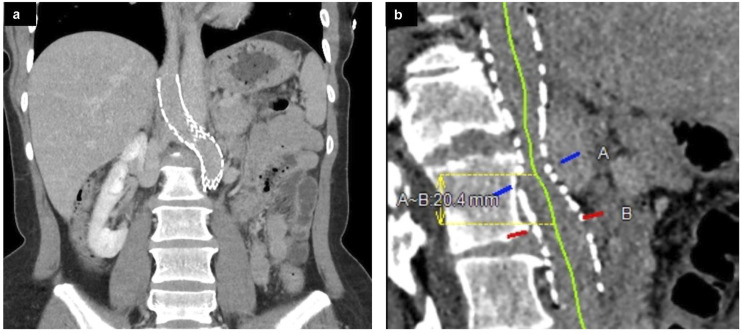
(A) Contrast-enhanced abdominal CT with coronal reformatted images obtained 2 months post-procedure demonstrates the course of the stent. (B) Curved multiplanar reformatted images demonstrate mild compression of the stented LIVC/renal vein behind the aorta with persistent patency.

At four-month follow-up, the patient reported a significant improvement in her lower extremity and pelvic venous symptoms including pelvic and lower extremity varices, but some mild intermittent back pain with certain activities suspected to be secondary to stent-related diaphragmatic irritation. Cross-sectional imaging at 2 and 4 month follow-up demonstrated mild compression of the retroaortic portion of the stent (Figure 7) with reduction in size of the varices in the upper thighs, abdominal wall, vulva, and deep pelvis, and right ovarian vein. Most notably, the patient successfully became pregnant approximately 3 months post-procedure, and is currently 38 weeks pregnant at time of authorship (approximately 1 year post-procedure). She was placed on prophylactic Lovenox for the duration of the pregnancy.

## Discussion

Left IVC, retroaortic LRV, and azygos continuation of the IVC have prevalences of .2-.5%, 2.1, and .6% respectively, and are typically asymptomatic incidental findings.^1,2^ While the combined prevalences of each of these anomalies can not necessarily be used to directly predict the rarity of a complex anomaly such as the 1 presented, the case is certainly a highly uncommon occurrence.

There are multiple prior reports of complex IVC anomalies in the case report literature. A 2019 case report described a patient with left IVC with hemiazygos continuation and drainage not into the azygos vein, but a left SVC with absence of the right SVC.^3^ Another 2019 report described a heterotaxic infant with left IVC and hemiazygos continuation with drainage into a duplicated SVC.^4^ Additional reports have detailed duplicated IVC with azygos continuation and retroaortic LRV, or the inverse case of duplicated IVC with hemiazygos continuation and retroaortic right renal vein.^1,5^ Two prior reports similar (but not identical) to the anomalous anatomy of the presented case were found; the reports described a left IVC with retroaortic crossing just above the LRV confluence to join the azygos vein.^6,7^ Importantly, both of those cases featured retroaortic courses of the infrahepatic IVC and ostensibly some degree of compression; however, none were symptomatic.

A third report from 2012 described a left IVC which passed anterior to the aorta after joining the LRV (the “classic” left IVC configuration), but which became compressed by the superior mesenteric artery resulting in a nutcracker syndrome with renal hypertension and hematuria. This case was treated with SMA transposition, but the authors suggested the possibility of endovascular intervention for future cases of left IVC compression.^8^ Accordingly, further literature search reveals that venous stenting is a known therapeutic option for LRV/SMA nutcracker syndrome and has been reported successfully for posterior LRV nutcracker syndrome, in which an anomalous LRV is compressed between the aorta and spine.^9,10^

This case report is limited primarily by a short follow-up period in a relatively young patient, with current imaging extending only to 4 months post-operative. As such, the durability and longevity of effectiveness of the retroaortic venous stent remains to be seen.

## Conclusion

The case presented here represents a rare complex IVC anomaly which was made even more extraordinary by the severe symptomatology experienced by the patient – most notably evidenced by pelvic congestion and recurrent miscarriages. It also represents, to our knowledge, the first documented instance of endovascular management of a left IVC/renal vein which crosses the midline (and is thus critically compressed) via a retroaortic course.

## Appendix

### Abbreviations

IVC: Inferior vena cava
SVC: Superior vena cava
LRV: Left renal vein
CEAP: Clinical, etiological, anatomical, pathophysiological (classification scheme for venous disorders)
SVP: Symptoms, varices, pathophysiology (classification scheme for venous disorders)
IVUS: Intravascular ultrasound
